# Successful conservative management of myiasis of an episiotomy wound and the uterine cavity postnatally: A case report

**DOI:** 10.1016/j.crwh.2025.e00709

**Published:** 2025-04-18

**Authors:** Satyala Satya Priya, Singuang Kamei Gaithaoliu, Kim Johanna Catharina Verschueren, Parishuddharao Koduri

**Affiliations:** a Department of Obstetrics & Gynaecology, Makunda Christian Leprosy and General Hospital, Bazaricherra, Karimganj, Assam, 788727, India; b Department of Obstetrics, Wilhelmina Children's Hospital, University Medical Center of Utrecht, Utrecht of University, Lundlaan 6, 3584 EA Utrecht, The Netherlands

**Keywords:** Myiasis, Maggots, Larvae, Genital, Intrauterine, Uterine cavity

## Abstract

Myiasis refers to an infestation by maggots or fly larvae. Urogenital myiasis, particularly in the uterine cavity, is extremely rare. Previously reported cases involved uterine prolapses, typically managed with hysterectomy. We describe the successful conservative management of myiasis in an episiotomy wound with extension into the uterine cavity. The case involved a woman in her 30s who gave birth to her third child eight days prior and presented with a painful and infected episiotomy site with “worms” emerging from her vagina. Her poverty had led to her malnourishment and poor hygiene; she was being treated for pulmonary tuberculosis. She required 11 days of inpatient care, which included broad-spectrum antibiotics, a three- to five-day course of ivermectin, clindamycin and albendazole, larvae extraction, manual vacuum aspiration, uterine cavity irrigation and the daily application of a menstrual pad soaked in turpentine oil. No further maggots were detected near the end of her hospital stay, nor at follow-up. This case demonstrates that conservative treatment can be effective, though it requires patience. Ensuring proper nutritional status and personal hygiene in the postpartum period is critical to preventing wound infections complicated by myiasis.

## Highlights

• Infected episiotomy wounds can result in myiasis in the uterine cavity.
• There is no guideline on the management of postpartum genital myiasis.
• Conservative treatment of intrauterine myiasis should be considered.
• Wound debridement, irrigation, larvae extraction, and antibiotics are effective.

## 1 Introduction

Myiasis, derived from the Greek word *myia* (“fly”), refers to the infestation of dipterous larvae (maggots) that feed on either living or necrotic tissue of a host [1]. The condition is commonly associated with poor hygiene, suboptimal general health, and the presence of wounds [2]. It occurs relatively frequently in rural and tropical regions across Africa, Asia, and the Americas [3]. The more common forms are cutaneous, nasopharyngeal, and ophthalmic myiasis, while genital myiasis is rare [4,5]. A literature search conducted in PubMed with the search terms “myiasis” and “uterine” identified few cases of internal genital myiasis involving the uterine cavity: five with uterovaginal prolapse and one case of a woman with a necrotic prolapsed fibroid postpartum. In all cases a hysterectomy was performed [[6], [7], [8], [9]]. There were no reported cases of myiasis in an infected episiotomy wound with migration into the uterine cavity, nor any cases of women with myiasis in the uterine cavity in which conservative management was successful.

## 2 Case Presentation

A married woman in her 30s presented with complaints of pain at her episiotomy site and reported the presence of “worms” dropping from her vulva. She had given birth to her third child eight days prior, during which an episiotomy was performed. During her pregnancy, she had attended four antenatal care visits, the first being in the mid-second trimester.

Upon her first presentation at 24 weeks of gestation, she complained of weakness, weight loss, fevers, and a chronic cough that had persisted for one month. She appeared malnourished (height 150 cm, weight 39 kg) and had severe anemia (Hb 4.5 g/dL). She was treated with albendazole and received two units of whole blood. She was clinically diagnosed with pulmonary tuberculosis and commenced empiric anti-tubercular treatment (ATT) for six months.

During the remainder of her pregnancy, she was advised to follow a high-protein diet and was supplemented with vitamin B complex, iron and calcium once daily. She attended further antenatal visits without additional complaints. At 39 weeks of gestation, she presented in spontaneous labor with meconium-stained amniotic fluid. Due to signs of fetal distress in a prolonged second stage of labor an episiotomy was performed. She gave birth to a healthy girl weighing 2540 g and both mother and baby were discharged after 24 h.

The patient lived in rural north-east India, in an area characterized by extreme poverty. Hygiene conditions around her home were poor, with no access to clean running water; open fields used for defecation and there were numerous flies in and around the house. Laundry was done in a nearby river, and drying clothes outside had been difficult due to the rainy season. She reported having to use damp cloths or to reuse the same cloths as sanitary pads for several days. Additionally, as part of a local ritual, she had not bathed since delivery, following the custom that postnatal mothers should abstain from bathing for the first 10 to 15 days.

**Fig. 1:**
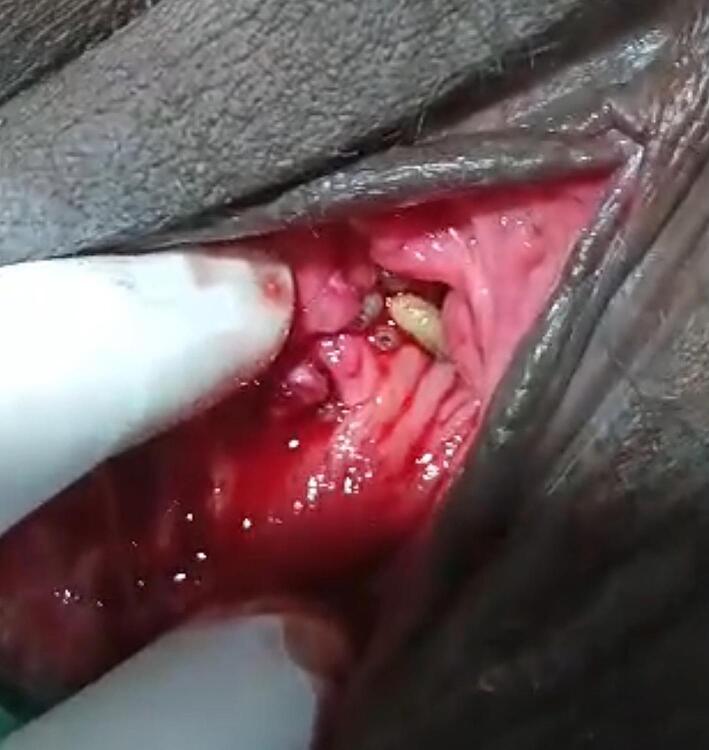
Infected episiotomy wound with intravaginal myiasis.

At the time of presentation, the patient had no additional complaints and seemed in normal mental and physical condition. Upon examination, her episiotomy wound was covered with sloughy tissue, and maggots were seen crawling in the wound and the vagina (Fig. 1). Laboratory investigations showed a hemoglobin level of 13.8 g/dL, a normal differential white blood cell count, and negative serology for HIV and syphilis. Routine urinalysis showed leukocytes (2+), some red blood cells (1+) and protein (1+), without any other abnormalities. Urine microscopy was unfortunately not performed.

The patient was admitted for wound care and started on broad-spectrum antibiotics (ceftriaxone 1000 mg once daily, metronidazole 500 mg three times per day and gentamicin 200 mg once daily) for five days. The wound was cleaned and disinfected twice daily with physiological saline 0.9 %. Maggots were manually removed when seen. Psychological support was provided. By the fourth day of admission, the episiotomy wound showed signs of improvement and granulation. However, the patient continued to see maggots during urination. A speculum examination revealed no maggots.

**Fig. 2:**
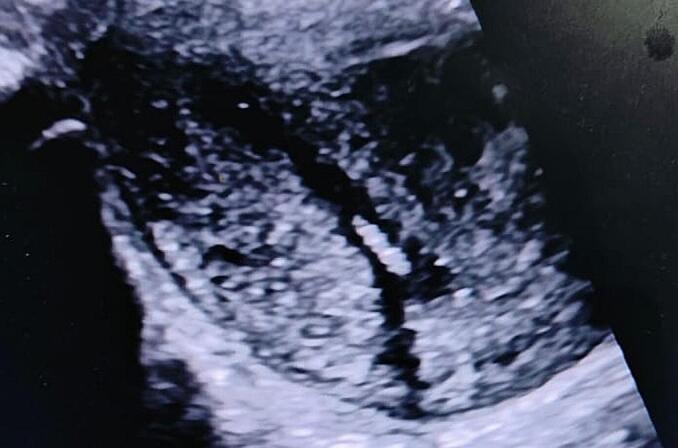
Sonographic image of maggots located low in the uterine cavity.

**Fig. 3:**
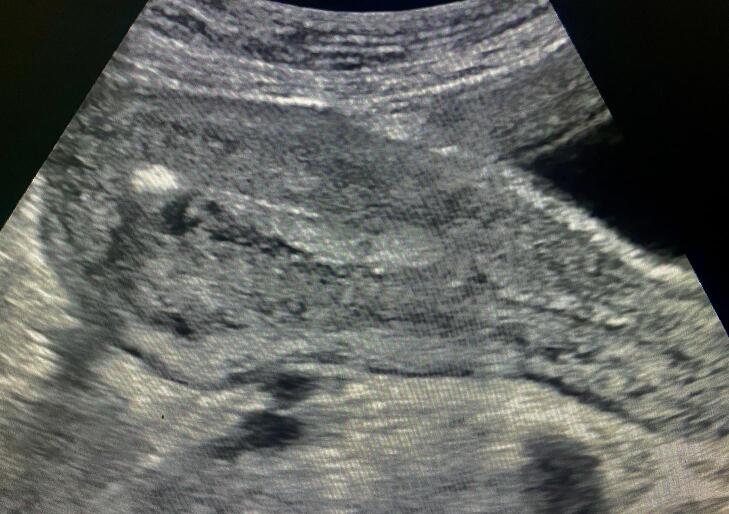
Sonographic image of maggots located high in the uterine cavity.

**Fig. 4:**
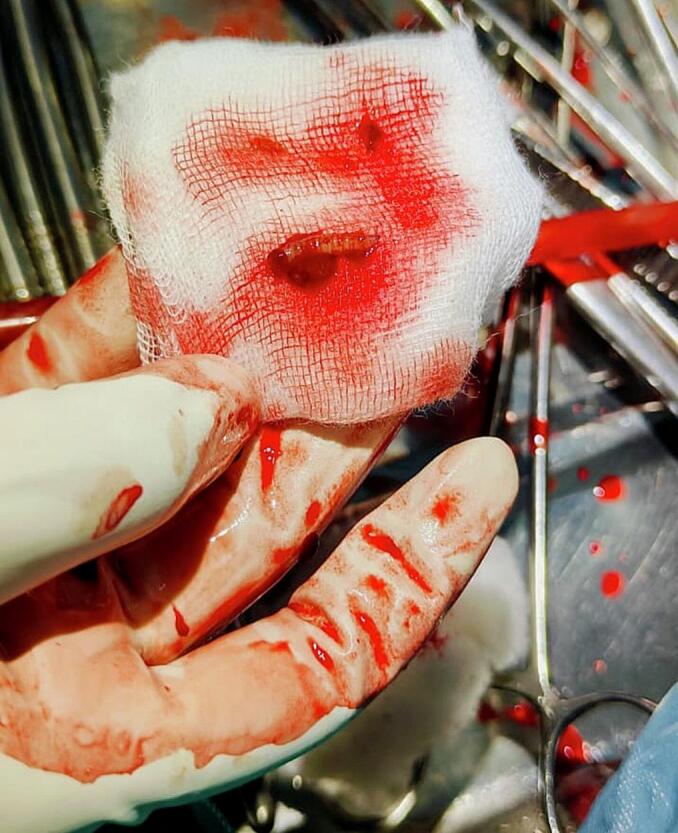
Maggot removed from the uterine cavity.

Sonography revealed a normal uterine cavity with several small hyperechoic fusiform masses (4 mm) in the cavity (Fig. 2, Fig. 3). These were confirmed to be maggots as several were removed under ultrasound guidance using Kelly uterine forceps (Fig. 4). Pain relief was provided with paracetamol 1000 mg and ibuprofen 400 mg. Unfortunately, some larvae were only partially removed, as they had anchored themselves into the endometrial tissue.

There was no local capacity to identify the specific larvae species [10]. The patient was started on a regimen of ivermectin 12 mg per day for three days and albendazole 400 mg twice per day for three days and clindamycin 300 mg three times per day for 5 days (a regimen based on study by Patel et al. [11]). A manual vacuum aspiration was performed to remove any necrotic tissue, possible retained products and remaining larvae. However, sonography still revealed several maggots. Following expert recommendations from Indian colleagues, daily lavage of the uterine cavity was conducted using a solution of hydrogen peroxide, metronidazole and sterile normal saline. The patient also used a pad soaked in turpentine oil in her underwear to attract the maggots.

**Fig. 5:**
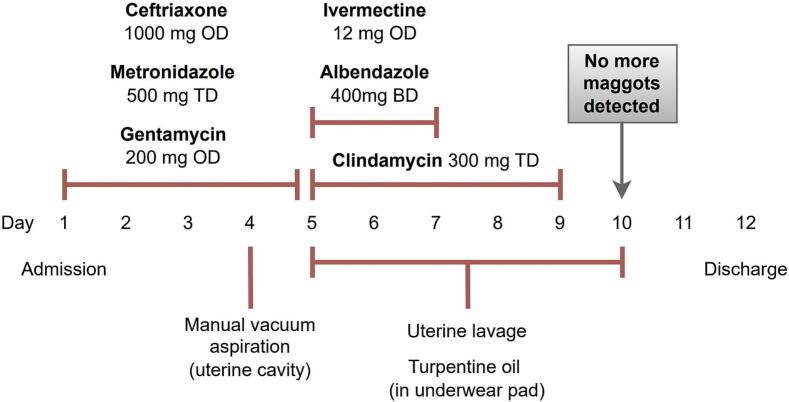
Timeline of treatment during admission.

The last maggot was observed five days after initiating the treatment regimen and lavage. After 48 h without further maggot detection, the patient was discharged after a total admission duration of 11 days. It is estimated that around 20 maggots were removed in total. Fig. 5 provides a timeline of the treatment. Before discharge, the patient and her family received counseling from social work on preventive measure to avoid reinfestation. These included proper wound care (regular cleaning and covering of the open wound), fly prevention (eliminating breeding sites with proper waste disposal, using mosquito nets, and maintaining a clean living environment), and, most importantly, improving personal hygiene (bathing and doing laundry with clean water) and good nutrition. At six-week follow-up, the episiotomy had healed, and the patient reported that she had seen no maggots since her discharge.

## 3 Discussion

Myiasis occurs when flies lay eggs in or on living or necrotic tissue. Most flies responsible for human myiasis belong to either the blowfly family (Calliphoridae) or the housefly family (Muscidae). Female flies are attracted to foul odor and deposit their eggs (typically 50–300 at a time) directly into a wound with necrotic or degenerative tissue, providing an ideal environment for the larvae to develop [12]. Alternatively, eggs may be laid on shaded soil, usually contaminated with urine or feces, or on drying clothes, such as improperly washed diapers or damp clothing [[1], [2], [3]]. At body temperature, the eggs hatch within 12–24 h, and the small larvae (approximately 1.7 mm long) penetrate living tissue. They release proteolytic enzymes and toxins, which break down surrounding living and necrotic tissue, which they feed on as they grow to their full size (7–9 mm). The larvae of all species have a tapered shape, with many rows of spines and hooks that are used to grip tissue [13]. Being photophobic, the maggots migrate deeper into tissue to hide in a protective niche, where they continue their development. After 5 to 7 days, they emerge from the tissue in search of a dry environment to pupate. Within 1–2 weeks, the adult fly emerges to complete the cycle. The larvae's photophobic nature explains their absence during speculum examination. Their shape and the presence of hooks accounts for the difficulty in removal. Furthermore, the duration of the life cycle, with the final stage of the maggots emerging after several days, highlights the need for patience when opting for conservative management [[1], [2], [3],^12^,^13^].

There are currently no established guidelines for the management and treatment of myiasis, particularly when the infestation extends into the uterine cavity. Commonly accepted approaches for managing wound myiasis include daily debridement, irrigation, and the application of turpentine oil to create a hypoxic environment, forcing the maggots to surface, which allows for their mechanical removal. Care should be taken not to rupture the larvae, as this could trigger a foreign body reaction, and to destroy the larvae after removal to prevent completion of their life cycle and re-infection [[13], [14], [15]].

Manual vacuum aspiration (MVA) is not the most effective method for removing intrauterine larvae. If there is certainty that no retained products are present in the cavity, it may be preferable to avoid MVA to prevent the risk of rupturing the larva and instead proceed directly with manual removal and lavage. Ultrasound-guided removal may be preferred over hysteroscopy, as the high-pressure retrograde flow of water during hysteroscopy could risk flushing the larvae into the abdominal cavity.

Although intrauterine irrigation for this specific indication has not been documented in the literature, in the present case it was likely most effective in creating a hypoxic environment that helped eliminate the larvae. However, special precautions are necessary when irrigating, as not all products (such as turpentine oil) can be used in the uterine cavity because of the high likelihood of abdominal spill through the tubes. The use of a hydrogen peroxide-metronidazole mixture for uterine irrigation was recommended by local experts. Although one study (Zhang et al.) supports the use of hydrogen peroxide, the potential complications, including subcutaneous emphysema and oxygen embolism, outweigh its benefits [15,16]. Therefore, in retrospect, we recommend against its use and instead advise using normal saline or Ringer's lactate.

Broad-spectrum antibiotics are often administered to treat or prevent secondary bacterial infections, although their efficacy in these cases remains uncertain [17]. In addition to mechanical removal, ivermectin is a viable treatment option, particularly when surgical removal is difficult, as it induces parasitic death and facilitates spontaneous expulsion [14]. Patel et al. suggest supplementing the ivermectin treatment with albendazole, an antihelminthic-antiparasitic agent that likely mobilizes parasites towards the surface, and clindamycin, a macrolide antibiotic that helps treat secondary infections and reduces the anaerobic conditions favorable to fly larvae [11].

While conservative management and irrigation for uterine cavity myiasis has not been reported in literature, previous cases of women with uterine myiasis concerned elderly women with uterine prolapse and one postpartum woman with a large prolapsed fibroid [2,4,[6], [7], [8], [9]]. We believe that episiotomy wound infections complicated by myiasis, with possible migration into the uterine cavity, may occur more frequently than previously thought and should be recognized as a neglected tropical disease [1]. Conservative management of uterine myiasis is effective and consists of the removal of larvae either manually or by irrigation, ivermectin, proper wound care with infection control and, finally, the prevention of reinfestation.
